# Use of Hand Creams during the Period of Frequent Disinfection in COVID-19 Pandemic—Preference Survey and Evaluation of Mercury Contamination

**DOI:** 10.3390/ijerph192013025

**Published:** 2022-10-11

**Authors:** Anna Puścion-Jakubik, Monika Pienkiewicz, Karolina Steckiewicz, Aleksandra Stypułkowska, Monika Grabia, Joanna Bielecka, Renata Markiewicz-Żukowska, Katarzyna Socha

**Affiliations:** Department of Bromatology, Faculty of Pharmacy with the Division of Laboratory Medicine, Medical University of Białystok, Mickiewicza 2D Street, 15-222 Białystok, Poland

**Keywords:** hand creams, mercury, SARS-CoV-2, questionnaire, safety of cosmetics

## Abstract

The skin is one of the ways the human body is exposed to toxic elements, including mercury (Hg). Hand creams are cosmetics that should be of high quality due to the fact that they can be used on irritated skin, which can facilitate the absorption of many ingredients. The study consisted of two stages: a consumer questionnaire was conducted regarding the preferences of the respondents, and then the Hg content in hand creams was determined. The survey covered 184 people with an age of 26.9 ± 7.8 years. Based on their preferences, 140 hand creams were selected for the study. The Hg content in the creams was determined by atomic absorption spectrometry with the amalgamation technique. The median content of this toxic element was 4.067 µg/kg. No influence of the country of origin, price, package size, main ingredients, and direction of action on the Hg content was shown. Noncarcinogenic risk assessment using the hazard quotient (HQ) indicated that the tested creams are safe. However, it should be emphasized that Hg has been shown in over 99% of the samples, which indicates that the content of this element in hand creams should be monitored.

## 1. Introduction

Mercury (Hg) is an element on which the attitude of the population has changed over the years. Initially, it was used in various areas of life, including medicine, industry, and pharmacy as well as beautification. Currently, the health risk resulting from its presence in the environment is known [1].

Hg exists as an inorganic and organic form. The term inorganic Hg includes metallic Hg, Hg vapors (Hg^0^), mercurous (Hg^2++^), and Hg salts (Hg^++^), whereas the term organic Hg refers to compounds in which Hg is associated with a structure containing carbon atoms (methyl, ethyl, or similar groups). Elemental Hg vapors are readily absorbed through the lungs and mucous membranes and then oxidize to other forms. Hg methyl, on the other hand, is easily digestible, e.g., through the intestines, but does not cross the blood–brain barrier as efficiently as elemental Hg—however, after passing to the brain, it is demethylated to elemental Hg. Hg salts are considered to be more stable, insoluble, and poorly absorbed [1,2].

Hg toxicity is influenced by factors such as the form of Hg, duration of exposure, and dose. Hg and its salts mainly damage the lining of the intestines and kidneys, methylmercury affects many systems and organs, while the brain is the target organ for Hg vapors [1,3]. Chronic exposure to Hg may manifest itself, among others, in skin rash [4].

The literature data indicates that increased Hg exposure, resulting in elevated blood Hg levels, is associated with an increased risk of nonmelanoma skin cancer (NMSC). This study was conducted among 29,413 people, whose average age was 49 years, and the data was collected from 2003 to 2016 [5].

The Sars-Cov-2 pandemic has changed many hygiene habits in the population. A study by Mościcka et al. showed changes in the frequency of using various cosmetics compared to the period before the pandemic. For example, about half of the respondents declared that they use hand creams as often during a pandemic as before the pandemic (48%), but 35% of people declared that they use slightly more, and 42% that they use much more [6].

The skin can be hairy or smooth. Smooth skin occurs on the soles of the feet and hands. This type is made of multilayer squamous epithelium, which makes the skin more resistant to various factors. Smooth skin, unlike hairy skin, does not contain sebaceous glands. For this reason, it requires special care [7].

The Hg content in cosmetics may be due to accidental contamination, but Hg can also be used as a preservative in eye products as thiomersal and phenylmercuric salts (including borate). The maximum permissible concentration of this element is 0.007%. In the case of deliberate addition of this type of substance, appropriate labeling on the product is necessary—it should be stated on the label that the product contains phenylmercuric compounds [8].

The aim of the study was to assess consumer preferences for hand creams, and then to assess the Hg content in selected creams, selected on the basis of criteria indicated by respondents.

## 2. Materials and Methods

### 2.1. Study Design

Our study was divided into 2 stages. In the first stage, an anonymous survey was conducted, which allowed to determine the preferences of users of hand creams. In the second stage, the quality of the creams was assessed in terms of Hg content.

### 2.2. Preference Questionnaire

The anonymous preference survey was promoted through social media. Answering all the questions was synonymous with the consent to participate in the study. The questionnaire contained 26 questions. The first part concerned the socio-economic assessment (age, sex, place of residence, social group, etc.), the next part concerned the frequency of disinfection, shopping preferences for cosmetics, with particular emphasis on hand creams, frequency of using creams and determining by respondents which cream brands are most often chosen by them. The individual questions are presented in Table S1.

### 2.3. Materials

The study included 140 hand creams, which are available online and stationary. When selecting the preparations for the research, their popularity was mainly taken into account (indicated by the respondents of the cream’s brand). These creams varied in terms of composition, the main direction of action indicated by the manufacturer, price, or country of origin of the product. Differentiation of the formulations was necessary to best assess the risk of Hg exposure. According to the manufacturers’ declarations, creams did not contain Hg or its compounds.

### 2.4. Methods

Hg content in hand cream samples was determined by atomic absorption spectrometry (AMA 254, Leco, Prague, Czech Republic). This method is based on the amalgamation technique. The measurement is performed directly, without prior digestion of the sample. The measurement consists of three steps: drying and burning the sample in a stream of oxygen (duration: 75 s), passing the released Hg vapors through the catalytic column, and capturing them by the amalgamator (duration: 150 s), then releasing Hg from the amalgamator and measuring its content at a wavelength of 254 nm (duration: 45 s). The Hg content in the sample was read on the basis of a standard curve prepared by diluting the standard Hg solution for atomic absorption spectrometry at a concentration of 1 g/L (Merck, Darmstadt, Germany). As a control of the accuracy of the method, the standard addition to the samples was used. Three measurements of Hg content were performed in each sample. The limit of quantification of the method used is 0.003 ng/sample.

### 2.5. Evaluation of Chronic Exposure to Hg

In order to assess the safety of the hand creams, the CDI (chronic daily skin intake) index was estimated. It was calculated according to the following formula:

CDI = $\frac{\mathrm{CS}\;\times \;\mathrm{SA}\;\times \;\mathrm{AF}\;\times \;\mathrm{ABS}\;\times \;\mathrm{EF}\;\times \;\mathrm{ED}\;\times \;\mathrm{CF}}{\mathrm{BW}\;\times \;\mathrm{AT}}$,

where:

CS—exposure-point concentration (result, mg/kg),

SA—exposed skin area (200 cm^2^),

AF—adherence factor (0.07 mg/cm^2^),

ABS—dermal absorption fraction (0.001, no units),

EF—exposure frequency (365 days/year),

ED—exposure duration (30 years),

CF—units conversion factor (10^−6^ kg/mg),

BW—body weight (70 kg),

AT—mean time for noncarcinogens (25,550 days) [9].

The HQ (hazard quotient) for Hg was calculated using the following formula [9]:

HQ = $\frac{CDIdermal}{RfDdermal}$,

where:

Reference dose (RfDdermal) for Hg: 0.0013 mg/kg/day [9].

If the HQ index is greater than 1, there is a potential risk to the health of the population; when the index is below 1, it is estimated that there is little risk that the population will experience side effects.

### 2.6. Statistical Analysis

Statistical analysis of the data was performed using the Statistica 13.3 program (Statsoft, Tibco, Palo-Alto, CA, USA). The normality of the data distribution was assessed using the Shapiro–Wilk, Kolmogorov–Smirnov, and Lilliefors tests. The Kruskal–Wallis test was used to show differences between several groups. At the level of significance, *p* < 0.05 was adopted.

## 3. Results

In our study, women were the vast majority (91.3%). Most of the residents came from small towns with up to 10,000 inhabitants (47.9%). Almost half of the respondents were students (46.7%) (Table 1). The average age was 26.9 ± 7.8 years.

The largest group were people who spent from 50 to 100 PLN monthly on cosmetic products. As many as 66.9% of the respondents described their economic situation as good, and 32.6% as average. The respondents indicated that in the last year they disinfected their hands more than 10 times a day (9.2%) or several times a day (52.2%). When analyzing commercially available hand creams, the respondents chose mainly creams containing ingredients of plant origin (29.4%) and oils (27.8%), while about one-third of the respondents did not pay attention to the composition (28.4%). The people surveyed most often chose creams with a price between PLN 10–30 (59.3%) or below PLN 10 (29.9%). Hand creams were most often applied 1–2 times a day (65.8%) or 3–4 times (19.6%). About 10% of the study group (9.2%) used creams more than 5 times a day (Table S2).

In the multiple-choice question, the highest percentage of respondents indicated cosmetics stores as the primary place to buy hand creams (84.2%). Pharmacies were ranked second (25.5%), and supermarkets third (21.2%). As many as 63.6% of respondents indicated that advertising did not affect their shopping preferences. The most important criteria guided by the respondents were: direction of action (62.0%), price (54.3%), smell (53.3%), consistency (38.0%), and composition (37.0%). The respondents most often bought the creams themselves (87.0%), depending on the need (44.1%). As many as 70.7% of respondents indicated that, in their opinion, more expensive creams are not better. The most preferred packaging was tubes (60.4%). About 3/4 of the respondents (74.5%) checked the expiry date of the creams. About 4/5 of the respondents (81.0%) read the description of creams (Table S2).

The main source of information about cosmetics for respondents is the internet (indicated by 82.1% of people), friends (50.5%), cosmetologists (23.4%), and studies (23.4%). According to the respondents, the most attractive form of promoting cosmetics is the low price (56.5%), free samples attached to other cosmetics (49.5%), and a second product for free (45.1%). Most respondents do not pay attention to whether the product has been tested on animals (52.2%). In addition, the respondents test different creams—only 5.4% declared the use of only one cosmetic brand, while as much as 89.2% used different brands (Table S2). The average amount of cream used once by the respondents was 3.0 ± 1.8 g.

About half of the hand creams tested came from Poland (52.2%). Most of the creams were cheap (60.7%), while 1/4 came from the so-called midpriced (25.7%). The first and main course of action in half of the creams was indicated by the producers of moisturizing (35.0%), then nourishing (18.6%), and regenerating (20.7%). The tested creams contained mainly ingredients of plant origin (34.3%) and oils (40.0%). Most of the creams were packaged up to 100 mL (51.4%) and 50 mL (45.7%) (Table S2).

The mean content of Hg in the tested creams was 6.666 ± 10.173 µg/kg (6.666 ± 10.173 × 10^−3^ mg/kg), while the median was 4.067 µg/kg (4.067 × 10^−3^ mg/kg). It was proved that the content of the main ingredients, the main course of action, country of origin, packaging volume, and price category had no influence on the Hg content in the creams. Due to the lack of statistically significant differences, only certain trends can be identified, which, however, require further analysis: the highest median Hg content was found in creams containing the so-called other ingredients (6.977 µg/kg), with a caring effect (11.058 µg/kg), originating from China (8.568 µg/kg), sold in packages with a capacity of up to 150 mL (5.932 µg/kg), or with the highest price (4.657 µg/kg), as shown in Table 2.

To assess the safe use of hand creams, the CDI was calculated followed by the HQ index. The CDI index was 5.720 × 10^−13^, while the HQ index was 4.400 × 10^−10^, which indicated that the tested hand creams do not pose a risk for long-term use.

## 4. Discussion

Washing hands frequently, especially during a pandemic period, may make hands coarser, irritated, and prone to infection. Research by Kampf and Ennen has shown that washing hands four times a day for 2 min leads to a gradual increase in hand skin roughness (from a starting level of 100 units to 108.5 units after 9 days). Regular use of hand creams reduces roughness (94.8 units after 14 days). The analysis of skin hydration showed a change resulting from hand washing from the initial level of 79 units to 65.5 units, while the use of creams allowed to increase this parameter to 75.6 units. This points to the fact that regular use of creams can prevent skin roughness and increase hydration [10].

Hand creams are a popular cosmetic. It is estimated that the value of the global market of hand creams and lotions will increase in 2022 compared to 2021 (from USD 5.73 mln to USD 6.43 mln) [11].

The COVID-19 pandemic indirectly contributed, inter alia, to increase the incidence of hand dermatitis in the group of nurses—it was reported in as many as 70.9% of people. Nurses, for disinfection, most often used liquid soap (55.8%), less often it was liquid soap and alcohol-based gel (21.2%), chlorhexidine-based gel (19.2%), and alcohol-based gel (3.8%). During the pandemic, this group experienced a significant increase in the frequency of hand washing, disinfection, and an increase in the frequency of using hand creams [12].

To the best of our knowledge, the content of Hg in hand creams has not been mentioned in scientific publications. Lightening creams were the group of cosmetics that underwent the most extensive safety assessment. The health risk related to their use was indicated over 20 years ago. For example, in Arizona, a study was conducted to determine the health effects of using a cosmetic cream containing Hg. The users of the cream had their urine Hg levels determined, and in the case of the elevated results in 66 people, further clinical evaluation was performed. Physical examination showed no abnormalities, although patients reported subjective neuropsychiatric symptoms [13].

The Hg content in a cosmetic may be influenced by many factors, including the purity of raw materials, individual stages of production, transport and storage, type of packaging, price, etc. Our research showed no effect of the price criterion on the Hg content in creams. Similar conclusions were made by Ho et al. [14], who analyzed the content of this element in 20 lightening creams. These creams were classified into four price categories. An interesting aspect is the fact that in cheaper creams, Hg was only shown in one sample: the average content was 80 ± 70 µg/kg. In the second price category, Hg was shown in 80% of the samples: the values ranged from 20 ± 20 to 640 ± 20 µg/kg. The highest result obtained in the third group was 1130 ± 110 µg/kg. In the group of the most expensive preparations, Hg was found only in one sample and its content was 60 ± 10 µg/kg.

According to the opinion of the Food and Drug Administration, Hg is not allowed in any other cosmetic products, apart from the eye products mentioned above. Its trace amounts, i.e., below 1 ppm (1000 µg/kg), are acceptable only when its presence is unavoidable and the product is obtained in accordance with good manufacturing practice (GMP) [15]. Regulation of Hg content in various products, including eye products and products intended for use on other areas of the body, vary from country to country. For example, in Japan, the presence of Hg is completely prohibited in both product categories. In China, preservatives are allowed in eye products, while for other cosmetics the norm is 1 ppm. In the USA, both product categories have a limit of 1 ppm. In European Union countries, it is allowed to use Hg as a preservative in eye preparations (the maximum permissible concentration is 0.007%), while its presence is prohibited in other cosmetics [16]. Our research showed that Hg was detected in practically all samples (over 99%), but the values were many times lower than 1000 µg/kg.

For example, Sin and Tsang studied Hong Kong residents (*n* = 314) who used creams. The Hg content exceeded the FDA standard many times, as it was as high as 660 to 57,000 ppm. Increased levels of Hg in the urine and blood were found in 65% of the study participants. Despite the fact that 78% of the people did not report any symptoms, it was not synonymous with low levels of Hg in blood and urine. The elevated Hg concentration in the blood was maintained only for 2 days after using the cream [17].

Skin lightening creams are used, among other reasons, to remove age spots or other pigmentation disorders. Al-Saleh et al. conducted studies on the safety assessment of these creams (*n* = 17). The concentration of Hg in the ovaries of mice was tested. The level in the control group was 11.70 ± 13.38 ng/g, while in the groups treated with creams: 2471.92 ± 1336.31 ng/g and 58.47 ± 39.51 ng/g. The authors concluded that skin exposure to Hg can cause accumulation in the ovaries of mice, which, with prolonged use, may contribute to infertility or ovarian failure [18].

It is not possible to define unequivocally what the source of contamination of creams with Hg may be. Presumably, the most important aspect is the high quality of raw materials used in production. However, contamination can occur at different stages in the marketing of a product. The form of Hg in the product is also important. The method used in our research allows the assessment of the total Hg content. The results obtained by us are low, within the normal range, and do not pose a significant health risk. However, it should be emphasized that Hg is a toxic element and long-term exposure to this element, from various sources, may lead to dangerous health consequences.

The ASA method with the amalgamation technique is one of the most frequently used methods to determine the Hg content in cosmetics, but also in biological samples or in food products. The AMA-254 Hg analyzer takes advantage of the ease with which Hg is released from its compounds, both inorganic and organic, into atomic form. The advantage of this method is that there is no need for prior microwave mineralization of the test, e.g., with nitric acid, i.e., no reagents, quick and easy execution. It can even detect trace amounts in the sample. Another method, used for the determination of Hg content, e.g., in surface waters, is the environmentally nontoxic terminating electrolyte [19].

Our study has several limitations. Hg content was determined in many samples of various origins. Perhaps the quality of creams sold at local markets, stored at inappropriate temperatures, would be different. Further research should be based on an assessment of the absorption of ingredients through the skin, including irritated and healthy skin, as well as through glabrous and hairy skin, to estimate the actual risk. These aspects should be taken into account by regulators establishing acceptable limits for the content of toxic components. In addition, the selection of samples for testing should reflect the percentage of production in each country.

## 5. Conclusions

Almost all tested hand creams had a Hg content above the detection limit. Due to the fact that in recent months the skin of the hands has been exposed to the repeated use of disinfectants during the day, its susceptibility to the penetration of toxic substances may be increased. Hand creams are applied to the skin of hands many times a day, so it is important to control their safety in terms of the content of impurities, including mercury content. Our research can be a significant suggestion for regulatory agencies and the cosmetics industry to control the quality of the product at every stage, as well as to randomly control products on the market.

## Figures and Tables

**Table 1:** Characteristic of study group.

Sex	*n* (%)
Woman	168 (91.3)
Man	16 (8.7)
**Place of residence**	
city up to 10,000 residents	88 (47.9)
city from 10 to 50 thousand residents	20 (10.7)
city with 50 to 100 thousand residents	18 (9.8)
city with over 100,000 residents	58 (31.6)
**Social group**	
Student	86 (46.7)
worker	35 (19.0)
white-collar worker	57 (31.0)
unemployed	6 (3.3)
retired pensioner	0 (0.0)

**Table 2:** Hg content in different categories of hand creams.

Division According to the Main Ingredient					
Categories	*n*	Av. ± SD (µg/kg)	Min–Max (µg/kg)	Med (µg/kg)	Q1–Q3 (µg/kg)
oils	56 (40.0)	5.993 ± 6.506	<dl–34.340	3.954	1.177–9.438
glycerin	8 (5.7)	5.705 ± 6.226	0.586–20.017	3.842	2.190–6.486
vitamins	6 (4.3)	8.635 ± 15.792	0.192–40.227	1.042	0.745–8.561
plant extracts	48 (34.3)	6.834 ± 13.897	0.151–86.921	3.491	0.843–5.700
bee products	7 (5.0)	9.317 ± 8.926	1.256–27.457	6.087	3.114–13.377
thermal water	3 (2.1)	3.823 ± 2.254	1.234–5.355	4.879	1.234–5.355
other	12 (8.6)	7.957 ± 8.663	0.118–33.507	6.977	3.572–8.593
**Division according to the main action**					
moisturizing	49 (35.0)	7.863 ± 13.564	0.239–86.921	4.744	1.161–7.482
regenerating	29 (20.7)	4.765 ± 4.502	0.118–15.318	3.404	1.064–7.215
caring	5 (3.6)	15.603 ± 16.927	0.745–40.227	11.058	1.060–24.864
protective	7 (5.0)	7.445 ± 11.976	0.8778–34.340	3.763	1.192–5.488
nourishing	26 (18.6)	5.770 ± 8.424	<dl–44.470	3.934	2.434–5.835
smoothing	14 (10.0)	5.023 ± 4.199	0.151–13.377	4.294	1.256–6.354
soothing	3 (2.1)	7.407 ± 3.825	3.054–10.231	8.936	3.054–10.231
other #	7 (5.0)	5.299 ± 7.038	0.192–20.017	1.979	0.812–7.669
**Division according to the country of origin**					
China	10 (7.1)	18.173 ± 25.404	1.979–86.921	8.568	5.333–20.026
Estonia	5 (3.6)	9.682 ± 19.466	0.305–44.470	0.809	0.394–2.434
Finland	12 (8.6)	5.476 ± 5.529	0.558–20.017	3.737	2.217–7.316
France	11 (7.9)	4.698 ± 5.097	0.118–18.949	4.434	1.003–5.104
Germany	9 (6.4)	3.929 ± 3.433	0.192–10.884	3.054	2.458–5.488
Poland	73 (52.2)	5.503 ± 6.598	<dl–40.227	3.855	1.161–7.669
USA	3 (2.1)	6.916 ± 6.638	0.270–13.545	6.934	0.270–13.545
Great Britain	10 (7.1)	6.509 ± 10.191	0.151–34.340	3.448	1.192–5.279
Other	7 (5.0)	8.966 ± 9.852	0.468–24.924	7.078	0.622–20.458
**Division according to the volume**					
up to 50 mL	64 (45.7)	7.512 ± 12.356	0.151–86.921	4.490	1.241–7.738
up to 100 mL	72 (51.4)	5.702 ± 7.686	<dl–44.470	3.483	1.078–7.349
up to 150 mL	4 (2.9)	10.470 ± 11.600	2.559–27.457	5.932	3.054–17.886
**Division according to the price**					
low	85 (60.7)	6.860 ± 11.431	<dl–86.921	3.895	1.161–7.154
average	36 (25.7)	5.816 ± 7.295	0.305–40.227	3.804	1.149–7.280
high	19 (13.6)	7.410 ± 9.154	0.269–34.340	4.657	1.479–7.215
Total	140 (100.0)	6.666 ± 10.173	<dl–86.921	4.067	1.177–7.576

Av.—average, dl—detection limit, IQR—interquartile range, Max—maximum value, Med—median, Min—minimum value, Q1—lower quartile, Q3—upper quartile, SD—standard deviation, no significant differences in the Kruskal–Wallis test. #—the term “other” includes toning, brightening, revitalizing, and energizing activities.

## Data Availability

Data are available from the authors.

## Supplementary Materials

The following supporting information can be downloaded at: https://www.mdpi.com/article/10.3390/ijerph192013025/s1, Table S1: Hand cream preference questionnaire; Table S2: Preferences of respondents regarding hand creams.
