# Supplementary material for: Use of Hand Creams during the Period of Frequent Disinfection in COVID-19 Pandemic—Preference Survey and Evaluation of Mercury Contamination
Source: Int J Environ Res Public Health. 2022 Oct 11;19(20):13025. doi: 10.3390/ijerph192013025 (PMC9602347; doi:10.3390/ijerph192013025)
Supplement: Supplementary file 1 [file ijerph-19-13025-s001.zip › ijerph-1925683-SM.pdf]

International Journal of
Environmental Research and Public Health

Supplementary Material

Use of Hand Creams During the Period of Frequent Disinfection in COVID-19 Pandemic - Preference Survey and Evaluation of Mercury Contamination

Anna Puścion-Jakubik *, Monika Pienkiewicz, Karolina Steckiewicz, Aleksandra Stypułkowska, Monika Grabia, Joanna Bielecka, Renata Markiewicz-Żukowska and Katarzyna Socha

Department of Bromatology, Faculty of Pharmacy with the Division of Laboratory Medicine, Medical University of Białystok, Mickiewicza 2D Street, 15-222 Białystok, Poland
* Correspondence: anna.puscion-jakubik@umb.edu.pl; Tel.: +48-8574-854-69

Table S1. Hand cream preference questionnaire.

Question	Answer
1. Age (years):	-
2. Gender:	Woman / Man
3. Place of residence:	city up to 10,000 residents / city from 10 to 50 thousand residents / city with 50 to 100 thousand residents / city with over 100,000 residents
4. Social group:	Student / worker / white-collar worker / unemployed / retired pensioner
5. What amount do you spend each month on the purchase of cosmetic products?	below PLN 20 / 20 - 50 PLN / 50 - 100 PLN / 100 - 200 PLN / over PLN 200
6. How do you evaluate the situation of your household?	good / mean / bad
7. How often did you use disinfectants in the last year?	a dozen or so times a day / several times a day / less than once a day
8. What ingredients of creams do you particularly prefer? If none of the above, in the answer "Other", please specify what:	oils / plant / bee / I do not pay attention to the composition / I don't use hand creams
9. Hand creams at what price do you most often use?	below PLN 10 / 10 - 30 PLN / over PLN 30 / I do not use hand creams
10. Please state how many creams (in grams) you use at one time. A large tube of cream contains about 100 g.	-
11. Please specify how many times during the day you used the hand cream:	1-2 / 3-4 / more than 5 / I do not use hand creams
12. Where do you buy your creams most often? You can mark more than 1 answer. If in any of the following, please provide a place in the "Other" field:	pharmacy / drugstore / Internet / supermarket / "catalog" sale / I do not use hand creams
13. Does advertising affect your shopping preferences?	yes / no / I do not use hand creams
14. Please specify the brands of the most commonly used hand creams:	-
15. What criteria are the most important for you when choosing a hand cream?	price / smell / package / action / volume / habit / mark / opinions of others / consistency / durability after opening / efficiency / ease of application / composition / I do not use hand creams
16. Who most often buys the cosmetics you use?	I buy myself / partner / siblings / parents / I do not use hand creams

17. How often do you buy hand creams?	1 x a week / 1 x a month / 1 x for 3 months / 1 x year / depending on the need / I do not use hand creams
18. Do you think that more expensive creams are better?	yes / no / I do not use hand creams
19. What kind of packaging do you prefer?	tube / dispenser with a pump / the jar / it does not matter to me / I do not use hand creams
20. Do you check the expiry dates of the cosmetics used?	yes / no / I do not use hand creams
21. Do you read the description of creams?	yes / no / I do not use hand creams
22. Where do you get your knowledge about cosmetics from?	studies / TV / Internet / work / friends / cosmetologist / pharmacist / I do not use hand creams
23. What forms of promotion of cosmetics do you prefer?	free samples included with the cosmetic / free samples included with the press / lower price / gift / discount coupon / the second product is free / I do not use hand creams
24. When choosing a hand cream, do you consider whether the product has been tested on animals?	yes / no / I do not use hand creams
25. Do you use only one brand of creams?	yes / no / I do not use hand creams
26. Comments, suggestions	-

Table S2. Preferences of respondents regarding hand creams.

**What amount do you spend each month on the purchase of cosmetic products?**	
categories	*n* (%)
below PLN 20	14 (7.6)
20 - 50 PLN	42 (22.8)
50 - 100 PLN	63 (34.2)
100 - 200 PLN	47 (25.6)
over 200 PLN	18 (9.8)
**How do you evaluate the situation of your household?**	
good	123 (66.9)
mean	60 (32.6)
bad	1 (0.5)
**How often did you use disinfectants in the last year?**	
a dozen or so times a day	17 (9.2)
several times a day	96 (52.2)
less than once a day	71 (38.6)
**What ingredients of creams do you particularly prefer? If none of the above, in the answer "Other", please specify what:**	
oils	51 (27.8)
plant	54 (29.4)
bee	12 (6.5)
I do not pay attention to the composition	52 (28.4)
glycerine, urea	1 (0.5)
shea butter	1 (0.5)
natural, with urea, with vitamins	1 (0.5)
niacamide, hyaluronic acid, vitamin A	1 (0.5)
panthenol, allantoin, urea	1 (0.5)
I don't use hand creams	10 (5.4)
**Hand creams at what price do you most often use?**	
below PLN 10	55 (29.9)
10 - 30 PLN	109 (59.3)
over PLN 30	10 (5.4)
I do not use hand creams	10 (5.4)
**Please specify how many times during the day you used the hand cream:**	

1-2	121 (65.8)
3-4	36 (19.6)
more than 5	17 (9.2)
I do not use hand creams	10 (5.4)
**Where do you buy your creams most often? (multiple choice question)**	
pharmacy	47 (25.5)
cosmetics shop	155 (84.2)
Internet	4 (2.2)
supermarket	35 (19.0)
"catalog" sale	19 (10.3)
supermarket	39 (21.2)
herbal and medical store	1 (0.5)
a small general store	1 (0.5)
I do not use hand creams	10 (5.4)
**Does advertising affect your shopping preferences?**	
yes	57 (31.0)
no	117 (63.6)
I do not use hand creams	10 (5.4)
**What criteria are the most important for you when choosing a hand cream?**	
price	100 (54.3)
smell	98 (53.3)
package	18 (9.8)
action	114 (62.0)
volume	40 (21.7)
habit	17 (9.2)
mark	17 (9.2)
opinions of others	41 (22.3)
consistency	70 (38.0)
durability after opening	7 (3.8)
efficiency	52 (28.3)
ease of application	41 (22.3)
composition	68 (37.0)
fast absorption	4 (2.2)
no feeling of stickiness	4 (2.2)
I do not use hand creams	10 (5.4)
**Who most often buys the cosmetics you use?**	
I buy myself	160 (87.0)
partner	4 (2.2)
siblings	2 (1.1)
parents	6 (3.3)
daughter	1 (0.5)
friend	1 (0.5)
I do not use hand creams	10 (5.4)
**How often do you buy hand creams?**	
1 x a week	1 (0.5)
1 x a month	41 (22.3)
1 x for 3 months	44 (23.9)
1 x year	7 (3.8)
depending on the need	81 (44.1)
I do not use hand creams	10 (5.4)
**Do you think that more expensive creams are better?**	
yes	37 (20.1)
no	130 (70.7)
it is difficult to say	2 (1.1)
not always	3 (1.6)
I pay attention to other features	2 (1.1)

I do not use hand creams	10 (5.4)
**What kind of packaging do you prefer?**	
tube	111 (60.4)
dispenser with a pump	28 (15.2)
the jar	2 (1.1)
it does not matter to me	33 (17.9)
I do not use hand creams	10 (5.4)
**Do you check the expiry dates of the cosmetics used?**	
yes	137 (74.5)
no	36 (19.6)
sometimes	1 (0.5)
I do not use hand creams	10 (5.4)
**Do you read the description of creams?**	
yes	149 (81.0)
no	23 (12.5)
sometimes	2 (1.1)
I do not use hand creams	10 (5.4)
**Where do you get your knowledge about cosmetics from?**	
studies	43 (23.4)
TV	18 (9.8)
Internet	151 (82.1)
work	12 (6.5)
friends	93 (50.5)
cosmetologist	43 (23.4)
pharmacist	19 (10.3)
family	1 (0.5)
doctor	1 (0.5)
internet blogs	1 (0.5)
I do not use hand creams	10 (5.4)
**What forms of promotion of cosmetics do you prefer?**	
free samples included with the cosmetic	91 (49.5)
free samples included with the press	15 (8.2)
lower price	104 (56.5)
gift	53 (28.8)
discount coupon	42 (22.8)
the second product is free	83 (45.1)
I do not use hand creams	10 (5.4)
**When choosing a hand cream, do you consider whether the product has been tested on animals?**	
yes	78 (42.4)
no	96 (52.2)
I do not use hand creams	10 (5.4)
**Do you use only one brand of creams?**	
yes	10 (5.4)
no	164 (89.2)
I do not use hand creams	10 (5.4)
